# The Impact of Coffee Consumption on Mortality Among Patients With Heart Failure

**DOI:** 10.7759/cureus.103926

**Published:** 2026-02-19

**Authors:** Mariana Alves, Filipe Dragovic, Catarina Correia, Tiago Rodrigues, M Rosario Oliveira, Daniel Caldeira

**Affiliations:** 1 Laboratory of Clinical Pharmacology and Therapeutics, Faculdade de Medicina, Universidade de Lisboa; Unidade de Ortogeriatria, Lisboa, PRT; 2 Instituto Superior Técnico, Universidade de Lisboa, Lisboa, PRT; 3 CEMAT and Department of Mathematics, Instituto Superior Técnico, Universidade de Lisboa, Lisboa, PRT; 4 Faculdade de Medicina, Universidade de Lisboa, Lisboa, PRT; 5 Centro Cardiovascular da Universidade de Lisboa – CCUL@RISE, CAML, Centro de Estudos de Medicina Baseado na Evidência (CEMBE), Laboratory of Clinical Pharmacology and Therapeutics, Faculdade de Medicina, Universidade de Lisboa, Lisboa, PRT; 6 Serviço de Cardiologia, ULS de Santa Maria, Lisboa, PRT

**Keywords:** caffeine, cardiovascular health, heart failure, mortality, nhanes, risk assessment

## Abstract

Aims: To assess the relationship between coffee consumption and all-cause mortality in heart failure (HF) patients, using data from the National Health and Nutrition Examination Survey (NHANES).

Methods and results: We analyzed data from NHANES (2003-2018), including 915 participants with HF who reported daily caffeine intake. Participants were categorized into coffee consumption levels: zero, one, two, three, and ≥4 cups/day. Multivariate logistic regression evaluated the relationship between coffee intake and mortality, adjusted for age, sex, and income. The mean participant age was 67, with 401 (44%) women. While one to three cups/day showed no significant association with mortality, consuming ≥4 cups/day increased mortality risk (OR: 1.58; 95% CI: 1.16-2.16; p=0.004). Age was the strongest predictor of mortality, while income and sex showed marginal associations.

Conclusions: Consuming up to three cups of coffee per day may be safe for patients with HF, while intake of four or more cups warrants caution due to the association with increased mortality. These findings underscore the need for further research to provide reliable recommendations.

## Introduction

Heart failure (HF) is a complex clinical syndrome characterized by high morbidity and mortality. Its prevalence continues to rise due to an aging population, an increasing burden of cardiovascular risk factors, and advances in therapeutic interventions, as well as improved survival rates. Recent data show that age-adjusted mortality in young adults (aged 15-44 years) increased from 2.36 in 1999 to 3.16 in 2019, a greater relative increase compared with older adults [1].

Regarding diet and lifestyle, coffee consumption is a frequently debated aspect of cardiovascular health [2]. Although extensively consumed and cautiously endorsed in hypertension and atrial fibrillation guidelines [3,4], the evidence concerning HF remains limited [5,6]. Previous cohort studies, covering European and American populations, suggest that caffeine intake does not increase the risk of developing HF, with some data even indicating an inverse association among women [7]. However, despite these findings on HF incidence, no large studies have analyzed the impact of coffee or caffeine specifically on the mortality of patients with established HF. Consequently, no specific recommendations exist regarding coffee intake for this population.

Therefore, to address this knowledge gap, our study aimed to investigate the association between coffee consumption and all-cause mortality in HF patients. Using data from the National Health and Nutrition Examination Survey (NHANES) [8], we also assessed the relationship between varying levels of coffee consumption and mortality outcomes in this population.

## Materials and methods

Study population

Data for this study were derived from the NHANES cycles conducted from 2003 through 2018. NHANES is a nationally representative survey that provides detailed information on the health and nutritional status of adults and children in the United States. Mortality outcomes for adult participants were obtained through linkage with the National Death Index (NDI).

The original study protocol was approved by the Ethics Review Board of the National Center for Health Statistics, and written informed consent was obtained from all participants. De-identified participant data are publicly available.

Variables

The population included in the study comprised individuals with self-reported HF who provided data on their coffee consumption (derived from the 24-hour dietary recall interview). To specifically assess coffee-related effects rather than total caffeine from all sources, participants were categorized based on their coffee consumption levels into five groups: non-coffee drinkers (zero), and those who drank one (one), two (two), three (three), or four or more (4+) cups per day. Demographic variables (age, sex, and income) were also recorded. Income levels were categorized into three groups: low, middle, and high.

The primary outcome of interest was all-cause mortality occurring during the study period.

Statistical analysis

To characterize the sample, descriptive statistics were computed. For categorical variables, frequency and sample distributions provided an overview of the sample’s demographic composition and coffee consumption levels. For continuous variables, the mean and standard deviation described the central tendency and variability of coffee intake and age.

The main focus of the analysis was a multivariate logistic regression, which modeled the likelihood of mortality as a function of coffee consumption levels, age, sex, and income. This regression model allowed for the assessment of each variable's effect on the risk of HF mortality while controlling for confounding factors. The coefficients from the regression were interpreted as odds ratios, indicating the relative odds of mortality associated with different levels of coffee consumption and demographic characteristics.

Statistical significance was assessed among categorical variables using Fisher’s Exact Test. This test was particularly useful for evaluating relationships within smaller sample sizes or when the assumptions of traditional chi-square tests were not met. A p<0.05 was considered statistically significant.

## Results

The initial sample size was 796,264. After excluding participants without HF (n=477,561) and missing data regarding caffeine intake and income (n=317,753), the number of remaining participants for analysis was 915.

Table 1 summarizes the characteristics of participants with HF. The mean age was 67 years, and 401 (44%) of the participants were women. The data were stratified by daily coffee intake, ranging from zero to 4+ cups per day.

**Table 1:** Baseline characteristics of participants. A p<0.05 was considered statistically significant. SD, standard deviation

	Number of cups of coffee per day						
	Overall	0	1	2	3	4+	p-value
n	915	385	404	93	24	9	
Mean age (SD)	66.85 (12.33)	63.28 (13.83)	69.10 (10.36)	71.34 (10.00)	68.38 (9.96)	67.78 (15.50)	<0.001
Female (%)	401 (43.8)	177 (46.0)	176 (43.6)	39 (41.9)	8 (33.3)	1 (11.1)	0.210
Mortality (%)	374 (40.9)	145 (37.7)	171 (42.3)	40 (43.0)	10 (41.7)	8 (88.9)	0.029
High income (%)	234 (25.6)	94 (24.4)	106 (26.2)	26 (28.0)	4 (16.7)	4 (44.4)	0.505

**Figure 1:**
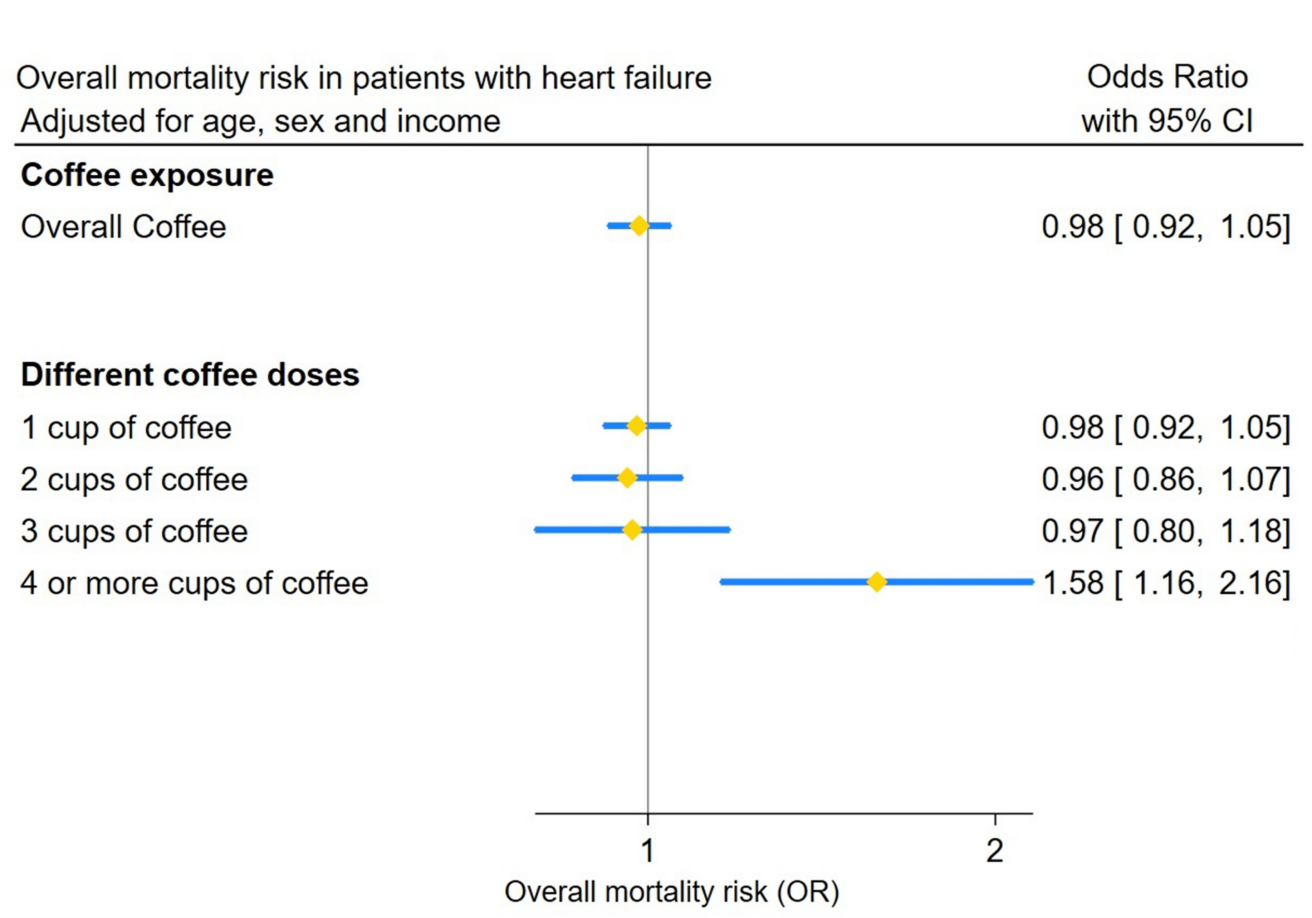
Overall mortality risk in patients with heart failure.

The relationship between coffee intake and mortality, before adjustment for confounders, showed that intake of coffee was associated with increased mortality rates (Table 1). Figure 1 shows the estimates for mortality risk adjusted for age, sex, and income. In HF patients, the overall coffee consumption was not associated with an increased risk of mortality (OR: 0.98; 95% CI: 0.92-1.05). However, consuming four or more cups of coffee daily was associated with an increased mortality risk (OR: 1.58; 95% CI: 1.16-2.16), while lower doses (one to three cups) showed no significant impact on mortality (Table 2 and Table 3 show the logistic regression model). It is important to note that the number of participants in the ≥4 cups category was small, which may affect the precision of this estimate.

**Table 2:** Logistic regression model summary with stratified coffee intake. A p<0.05 was considered statistically significant (*); **p<0.01; ***p<0.001. SE, standard error

Predictor	Estimate	SE	t-value	p-value	Significance
(Intercept)	-0.331	0.087	-3.785	<2e-4	***
1 cup of coffee	-0.022	0.034	-0.637	0.524	-
2 cups of coffee	-0.041	0.056	-0.742	0.459	-
3 cups of coffee	-0.031	0.099	-0.314	0.754	-
4+ cups of coffee	0.457	0.159	2.878	0.004	**
High income	-0.068	0.036	-1.904	0.057	.-
Female	-0.047	0.031	-1.479	0.139	-
Age	0.012	0.001	9.058	<2e-16	***

**Table 3:** Logistic regression model summary with binary coffee intake. A p<0.05 was considered statistically significant. SE, standard error

Predictor	Estimate	SE	t-value	P-value
(Intercept)	-0.324	0.087	-3.702	<0.001
1 cup of coffee or more	-0.017	0.032	-0.530	0.596
High income	-0.064	0.036	-1.791	0.074
Female	-0.052	0.031	-1.654	0.098
Age	0.012	0.001	8.980	<0.001

## Discussion

Our study suggests that consuming up to three cups of coffee per day may be safe for patients with HF, while consuming four or more cups was associated with increased mortality.

This finding supports the hypothesis that excessive caffeine intake may stress the cardiovascular system [9]. Indeed, a J-curve phenomenon has been reported in systematic reviews evaluating the risk of incident HF and other cardiovascular events associated with coffee [2,10].

Previous studies, including the Framingham Heart Study (FHS), Cardiovascular Health Study (CHS), and Atherosclerosis Risk in Communities (ARIC) study, reveal that higher coffee consumption was associated with lower long-term risk of HF. A statistically significant J-shaped relationship was observed, suggesting that up to four servings of coffee per day could be protective for incident HF. Nonetheless, at extreme coffee consumption (around nine to 10 servings per day), the HF risk appears to increase [11].

However, it is crucial to distinguish between preventing HF in the general population and managing outcomes in patients with established disease. While previous large-scale cohorts, such as the FHS and ARIC, have established that habitual coffee consumption of up to four cups daily is associated with a reduced risk of incident HF, our findings indicate a divergent outcome for mortality in patients with established HF [12]. This discrepancy highlights a critical distinction between prevention and prognosis. In a healthy cardiovascular system, the intake of three to four cups/day represents the optimal window for mortality reduction, with benefits plateauing or diminishing at higher intakes [13]. However, in the failing heart, the physiological reserve to handle the acute hemodynamic effects of higher caffeine doses, such as transient tachycardia and increased catecholamine release, may be compromised [11]. Thus, the "J-curve" phenomenon frequently described in cardiovascular epidemiology appears to shift toward a lower threshold in this vulnerable population, turning a potentially protective volume (four cups) into a risk factor [14].

Additionally, findings from the Coronary Artery Risk Development in Young Adults (CARDIA) study indicated that low to moderate coffee consumption from young adulthood through middle age was associated with improved left ventricular systolic and diastolic function, highlighting potential long-term cardiovascular benefits of moderate coffee intake [15]. Nevertheless, extrapolating these benefits to a population with established cardiac dysfunction requires caution.

Several studies have demonstrated an association between moderate coffee consumption and reduced all-cause and cardiovascular mortality. One meta-analysis identified the lowest risk of all-cause mortality at an intake of approximately 3.5 cups per day (RR: 0.85; 95% CI: 0.82-0.89) [16]. Another study reported that individuals consuming at least four cups of coffee daily exhibited a 64% lower risk of all-cause mortality compared to non-coffee drinkers [11]. However, these findings may not be directly extrapolated to HF patients, necessitating dedicated investigations into the effects of caffeine in this specific population.

Notably, in atrial fibrillation, an arrhythmia that can both trigger or lead to HF, caffeine intake has not been shown to significantly increase risk [17]. It is also important to emphasize that in patients with coronary artery disease, which is an important cause of HF, particularly those who had a myocardial infarction, higher doses of coffee consumption have not been associated with adverse cardiovascular events [18].

A small randomized controlled trial confirmed the acute safety of high doses of caffeine in patients with HF [19,20]. However, to our knowledge, no studies to date have specifically examined the impact of coffee on mortality in patients with HF. Our findings provide a preliminary step in understanding this relationship, highlighting the need for larger, well-designed studies to elucidate the effects of caffeine on HF outcomes.

This study has several strengths, including the use of a diverse participant sample and adjustment for key demographic variables, improving the generalizability of our findings. However, limitations regarding these findings must be acknowledged. First and foremost, reliance on a single 24-hour dietary recall for caffeine intake assessment introduces potential recall bias and may not reflect long-term habitual consumption. Second, the reliance on self-reported HF diagnosis may have introduced misclassification bias. Importantly, the small sample size in the highest coffee consumption category (≥4 cups) is a key limitation that may have reduced statistical power and precision; therefore, the observed increased risk in this specific subgroup should be interpreted with caution.

Future research should explore larger, longitudinal cohorts to assess the impact of sustained coffee or caffeine consumption on HF outcomes. To strengthen causal inference, future studies should move beyond single 24-hour recalls and incorporate multiple dietary assessments or use biomarkers of caffeine intake (e.g., plasma or urinary metabolites). Investigating caffeine sources beyond coffee may also clarify whether sources of caffeine differentially affect HF prognosis. Additionally, future studies should incorporate more precise methods for diagnosing HF, such as clinical assessments and biomarker-based evaluations, to mitigate the limitations associated with self-reported HF diagnosis.

## Conclusions

In conclusion, based on this observational analysis, our findings indicate that moderate coffee consumption, up to three cups per day, was not associated with increased mortality in patients with HF. In contrast, higher levels of consumption (four or more cups daily) were associated with potential adverse outcomes; however, this finding must be interpreted with caution due to the limited sample size of the high-intake subgroup. These results highlight the importance of individualized dietary counseling in patients with HF, taking into account overall cardiovascular risk, comorbidities, and tolerance to caffeine. Ultimately, to definitively establish causality and determine precise safety thresholds, further high-quality prospective studies and randomized clinical trials are needed before robust, evidence-based recommendations can be issued.
