# Correction: The Impact of Coffee Consumption on Mortality Among Patients With Heart Failure

**DOI:** 10.7759/cureus.c420

**Published:** 2026-03-27

**Authors:** Mariana Alves, Filipe Dragovic, Catarina Correia, Tiago Rodrigues, M Rosario Oliveira, Daniel Caldeira

**Affiliations:** 1 Laboratory of Clinical Pharmacology and Therapeutics, Faculdade de Medicina, Universidade de Lisboa; Unidade de Ortogeriatria, Lisboa, PRT; 2 Instituto Superior Técnico, Universidade de Lisboa, Lisboa, PRT; 3 CEMAT and Department of Mathematics, Instituto Superior Técnico, Universidade de Lisboa, Lisboa, PRT; 4 Faculdade de Medicina, Universidade de Lisboa, Lisboa, PRT; 5 Centro Cardiovascular da Universidade de Lisboa – CCUL@RISE, CAML, Centro de Estudos de Medicina Baseado na Evidência (CEMBE), Laboratory of Clinical Pharmacology and Therapeutics, Faculdade de Medicina, Universidade de Lisboa, Lisboa, PRT; 6 Serviço de Cardiologia, ULS de Santa Maria, Lisboa, PRT

This article has been corrected to update the institutional affiliations of all authors as the originally published version listed incorrect affiliations.

